# The effect of body mass index on the risk of surgical site infection

**DOI:** 10.1186/2047-2994-4-S1-O29

**Published:** 2015-06-16

**Authors:** AP Meijs, SC de Greeff, MC Vos, SE Geerlings, MB Koek

**Affiliations:** 1 Centre for Infectious Disease Control, National Institute for Public Health and the Environment (RIVM), Bilthoven, Netherlands; 2 Department of Medical Microbiology and Infectious Diseases, Erasmus MC, Rotterdam, Netherlands; 3 Division of Infectious Diseases, Department of Internal Medicine, Academic Medical Center, Amsterdam, Netherlands

## Introduction

Body mass index (BMI) is considered a risk factor for surgical site infections (SSIs).

## Objectives

In this study we quantified the impact of BMI on the risk of SSI for a variety of surgical procedures.

## Methods

We included data on SSIs collected in 2012 and 2013 in the Dutch surveillance network PREZIES. A selection of frequently performed surgical procedures across different specialisms was made: laparoscopic cholecystectomy, open colectomy, laparoscopic colectomy, abdominal hysterectomy, Caesarean section, mastectomy, lumpectomy, total hip prosthesis and total knee prosthesis. Patients were stratified into five BMI categories: underweight (BMI < 18.5), normal weight (BMI 18.5-25 kg/m^2^), overweight (BMI 25-30 kg/m^2^), obese (BMI 30-40 kg/m^2^) and morbidly obese (BMI >40 kg/m^2^). Multilevel log binomial regression analyses were performed to assess the effect of BMI on the risk of SSIs while accounting for clustering within hospitals.

## Results

Of the 62,647 included patients (ranging from 1,445 for abdominal hysterectomy to 18,575 for total hip prosthesis), 1% were underweight, 31% had normal weight, 40% were overweight, 26% had obesity and 2% were morbidly obese. SSI incidence varied from 1.1% for total knee prosthesis to 17.4% for open colectomy. Obese patients had an increased risk of SSI compared to the normal weight (reference) group for lumpectomy (relative risk [RR] =3.0), total hip prosthesis (RR=2.9), mastectomy (RR=2.0), laparoscopic colectomy (RR=1.5) and open colectomy (RR=1.3) (all p-values <0.02). The risk of SSI in the morbidly obese group was significantly increased for abdominal hysterectomy (RR=7.6), lumpectomy (RR=7.4), total hip prosthesis (RR=6.9), Caesarean section (RR=3.8), laparoscopic cholecystectomy (RR=2.9), mastectomy (RR=2.7), total knee prosthesis (RR=2.6) and open colectomy (RR=2.0). Underweight resulted only for mastectomy in an increased risk of SSI (RR=2.6; 95% CI 1.1-5.8).

## Conclusion

We found that for most procedures obese and morbidly obese patients had an at least 1.3 times increased risk of SSI compared to normal weight patients, whereas the increased risk of SSI for underweight patients was less profound. As a result, the ever expanding prevalence of obesity will take on an increasingly important role in the prevention of SSI.

## Disclosure of interest

None declared.

